# Incidental Rickets in the Emergency Department Setting

**DOI:** 10.1155/2012/163289

**Published:** 2012-10-09

**Authors:** John V. Zurlo, Shaun R. Wagner

**Affiliations:** Department of Radiology, Saint Barnabas Medical Center, 94 Old Short Hills Rd, Livingston, NJ 07039, USA

## Abstract

Vitamin D deficiency rickets is a childhood osteomalacia, with impaired skeletal development and potentially skeletal deformities. The radiographic findings of rickets are many but include widening, fraying, and cupping of the metaphysis. Developmental delay and related complications of seizure and tetany have also been reported. This medical entity is often thought of as a classic medical disease of the past. However, it persists, and the recognition of rickets is on the rise. The reemergence of rickets correlates with the increase in the number of children exclusively breastfed and with the frequent use of sun block in the pediatric population. We present two cases of rickets, diagnosed through a visit to the Emergency Department made for unrelated symptoms. These two cases illustrate the importance of diagnosing rickets as an “incidental” finding. With early detection, dietary supplementation can be initiated potentially sparing the patient symptomatic disease.

## 1. Introduction

Rickets is a failure of bone mineralization. Vitamin D deficient rickets in the developed world is rare but its recognition is increasing. Rickets can lead to bone deformities as well as developmental delay and related complications of seizure and tetany. Links have also been made between vitamin D deficiency and a predisposition to respiratory infections during infancy [1, 2].

The active form of Vitamin D is 1,25 (OH_2_) D3 (calcitriol). This active form functions as a hormone, acting upon many systems within the body and is derived from the hepatic and renal metabolism of Vitamin D3 (cholecalciferol). Vitamin D3 may be directly sourced from dietary intake, derived from dietary supplements or produced in the skin with ultraviolet light exposure from precursors [1, 3].

Patients may present to the Emergency Department (ED) with unrelated or nonspecific complaints resulting in plain film and laboratory evaluation. Consequently, Emergency Department physicians and radiologists should be aware of the radiographic and laboratory signs of this disease and be vigilant in their detection. In the emergency room setting, nutritional rickets may be suggested by decreased serum calcium and phosphorus in conjunction with increased serum alkaline phosphatase. Radiographic findings of rickets are many but include widening, fraying, and cupping of the metaphyses. Findings are most pronounced at the ribs, knees, and wrists. In children who bear weight, there may be bowing of the tibia and in children who crawl, bowing of the upper extremity [3]. Radiographic evaluation plays a considerable role in diagnosis of clinically silent or subtle rickets. 

## 2. Case Reports

### 2.1. Case 1

 An 8-month-old African American male presented to the ED with his mother; the chief complaint was fever (reported *T*_MAX_ 102.3F). The mother also reported crankiness and decreased oral intake. Review of the patient's history revealed that he had been almost exclusively breastfed. Additionally, the patient had not received any medical care since 4 weeks of age. 

**Figure 1:**
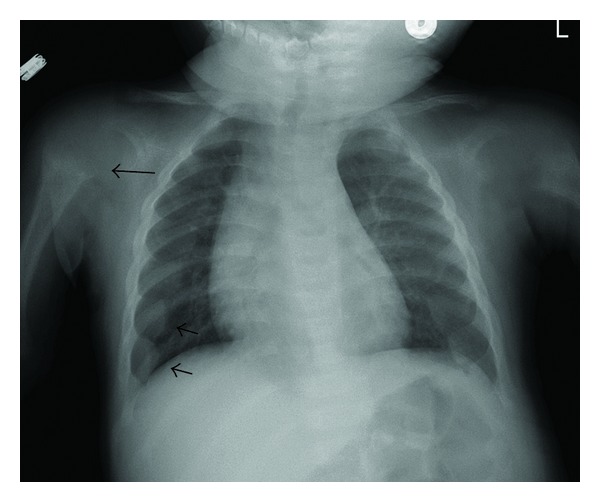
Chest radiograph of an 8-month-old male with fever demonstrating widened anterior ribs (short arrows) and proximal humeral metaphyseal fraying and cupping (long arrows). (Reprinted with permission of the American College of Radiology (ACR's Case in Point, March 20, 2012, Rickets). No other representation of this material is authorized without expressed, written permission from the American College of Radiology).

While in the Emergency Department, the patient underwent plain film exam of the chest for potential pneumonia. The Emergency Department physician initially interpreted the exam as normal. He was discharged and instructed to follow up with a primary care physician. Subsequently, the films were reviewed by a radiologist who noted flaring of the ribs at the costochondral junction (“rachitic rosary”), as well as fraying and cupping of the proximal humeral metaphyses bilaterally (Figure 1). The ED physician was notified of these findings, and the patient was recalled for further evaluation.

**Figure 2:**
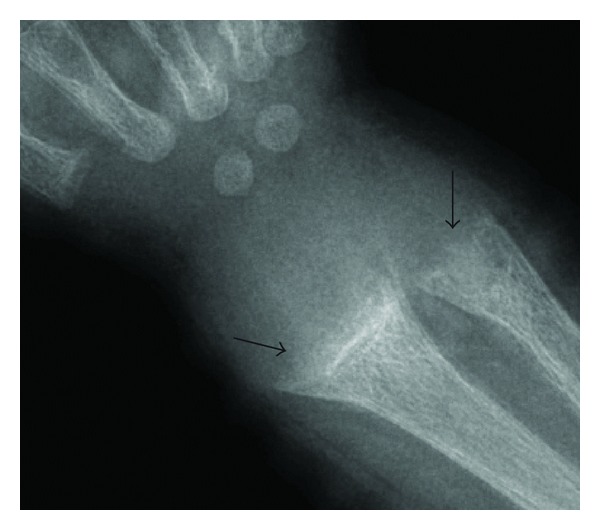
Wrist radiograph of the same patient as Figure 1. The distal metaphysis of the radius and ulna is widened and frayed with a mild cupped configuration (arrows).

Laboratory work drawn at the patient's return demonstrated normal serum calcium levels, markedly elevated parathyroid hormone and alkaline phosphatase as well as low phosphorus. Laboratory values for calcidiol (25-OH vitamin D3) were below threshold for detection. Wrist films were taken and demonstrated fraying and cupping of the distal radial and ulnar metaphyses (Figure 2). Given the radiographic findings and abnormal lab values, the patient was admitted to the pediatric service with the diagnosis of vitamin D deficiency rickets. He was treated with vitamin D 2000 IU orally daily for the duration of admission. The mother was educated in regard to proper nutrition and instructed to supplement the patient's diet with 400 IU vitamin D daily.

### 2.2. Case 2

A 17-month-old African American female presented to the Emergency Department with her parents reporting that the patient was vocally distressed when any degree of pressure was applied to the her chest, especially when picked up. The parents also report that the patient had not been using her right arm or rolling over as she was accustomed. Further inquiry revealed that the patient had been “knocked over” from the seated position by a sibling shortly before these symptoms started. Past medical history was unremarkable. Assessment of developmental milestones revealed that the patient had not yet started to walk. Otherwise, the expected milestones had been met.

**Figure 3:**
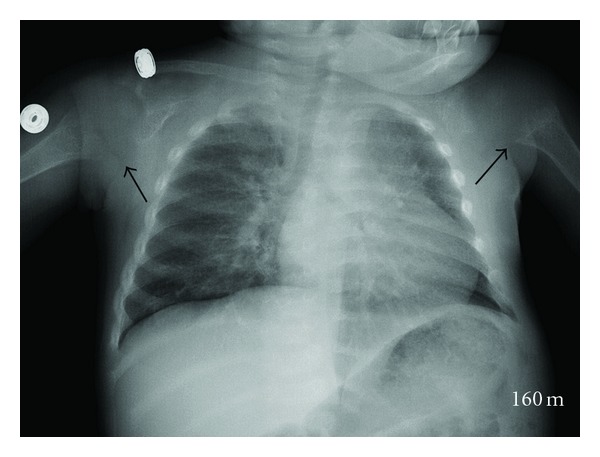
Chest radiograph of a 17-month-old female with pain and recent trauma. The proximal metaphyses of each humerus are irregular with widening (long arrows).

The patient underwent plain film examination of the chest (Figure 3). Evaluation revealed abnormal proximal humeral metaphyses bilaterally. A subtle lucency was questioned in the right clavicle prompting plain film evaluation of the clavicles and upper extremity. These films revealed no fracture. However, abnormalities were demonstrated at the proximal metaphysis of the humerus as well as at the distal metaphyses of the radius and ulna. The bones appeared osteopenic. 

The patient's pediatrician subsequently referred the child to a pediatric endocrinologist. The outpatient workup was not part of the patient's hospital medical record, but she was reported to have been diagnosed with nutritional Vitamin D deficiency and treated with vitamin supplements.

## 3. Discussion

The two cases illustrate that rickets is not a disease of the past. Incidental cases of rickets presenting through the Emergency Department have been reported in the emergency medicine literature [2, 4]. Emergency Department physicians and radiologists reading emergent films must follow a set search pattern that attends to all portions of the provided image, not just those pertaining to the chief complaint. Particularly when reviewing pediatric films, it is important to remain vigilant for radiographic signs suggestive of rickets. Failure to recognize rickets can lead to complications including progressive bone deformities and related hypocalcemia complications of seizures, tetany, abnormal electrocardiograms, and pneumonia [2].

Rickets is fundamentally a failure of mineralization of osteoid. Maintenance of serum calcium levels is dependant on vitamin D. Vitamin D has many physiologic effects but its key effect is its ability to promote gastrointestinal absorption of calcium. The active form of Vitamin D is 1,25 (OH_2_) D3 (calcitriol). Calcitriol is produced from hepatic and renal metabolism of vitamin D3 (cholecalciferol). Vitamin D3 can be produced endogenously from previtamin D3, which in turn is produced from cholesterol metabolites in the skin with ultraviolet light (sunlight). Vitamin D3 can also be supplied directly from diet or supplements [1, 3].

With appropriate levels of serum calcium and phosphorus, there is normal maturation of the three layers of chondrocytes of the growth plate: the reserve zone, proliferating zone, and hypertrophic zone. In rickets, the growth plate continues to grow but in an disorganized fashion. There is a failure of osteoblast replacement of chondrocytes and ultimately a failure of ossification [3].

The earliest specific radiographic finding of rickets is widening of the growth plate. Cupping of the long bone metaphysis as well as enlargement of the costochondral junction (rachitic rosary) develops later and is characteristic. In addition, the metaphysis may appear frayed. Metaphyseal findings are most pronounced at sites of rapid growth: costochondral junction of the middle ribs, distal femur, proximal humerus, proximal and distal tibia, and distal radius and ulna. Widening reflects physeal growth without ossification; fraying reflects disorganization of the growth plate. Trabeculae will appear course, and the bones will be osteopenic. Epiphyseal ossification may be delayed. Infants may develop a craniotabes, a squared skull configuration. Bowing of the long bones may be present, as the bones soften due to lack of adequate mineralization. However, bowing requires some degree of weight bearing; in an infant, bowing may be present at the long bones of the upper extremities due to crawling, whereas an older child is more likely to exhibit bowing of the legs. Scoliosis and other deformities may occur [3].

Rickets should be diagnosed with a combination of radiographs and laboratory findings. Nutritional rickets commonly results in normal to decreased serum calcium, as well as normal to elevated serum phosphorus. There are decreased levels of calcidiol (25 (OH) vitamin D3) and urinary calcium. Levels of parathyroid hormone, alkaline phosphatase, and urinary phosphorus are typically elevated [1]. 

Recent literature has documented cases of unusual fractures that were originally confidently diagnosed by radiograph as nonaccidental injury [5]. However, subsequent laboratory evaluation revealed findings consistent with rickets, exonerating the parents from suspicion of abuse. When assessing a pediatric patient for risk of abuse, it is important to be as thorough as possible in exam and workup. It is not acceptable to return a child to an unsafe environment nor place caring parents under undue suspicion and unnecessarily remove their child from their care. To start, the physician should perform a detailed review of the circumstances surrounding the injury, including how the injury occurred, child's dietary history, the mother's diet during pregnancy, and developmental history (rolling, walking, running, climbing, etc.). Assessment should be made of the child's risks for abuse and maltreatment. Family history may reveal patterns of skeletal disease such as short stature or fractures. When suspicion of abuse arises, the American Academy of Pediatrics recommends performing a skeletal survey on children as old as 2 years to evaluate for occult and/or nondisplaced fractures. This survey is to be followed up in two weeks to evaluate for healing fractures that may not have been apparent on the initial imaging. The probability of rickets going unnoticed radiographically is very low when these skeletal surveys are performed correctly [6]. 

Rickets is most commonly caused by a nutritional deficiency in vitamin D. However, other causes include errors in metabolism of vitamin D, nutritional deficiencies in calcium or phosphorus, and renal phosphate wasting. Some medications can contribute to rickets. Modern lifestyles predispose those living in developed countries to vitamin D deficiency. The vast majority of vitamin D is produced endogenously through biochemical reactions involving UV-B light. Studies have shown that adults spent up to 93% of their waking hours indoors, with similar smaller studies indicating children spend roughly the same percentage of time indoors. That limited amount of sun exposure is not sufficient to produce the amount of vitamin D necessary to sustain adequate bone mineralization. Furthermore, the push toward sunscreen use for prevention of skin cancer limits the amount of vitamin D produced when outdoors. Sunscreens block the UV-B rays necessary to convert 7-dehydrocholesterol into previtamin D3. Dietary availability is quite limited, being found in significant quantities only in fatty fish, fish oils, egg yolks of chickens fed vitamin D, and fortified milk [1, 7]. 

Infants that are exclusively breastfed are at a greatly increased risk of developing vitamin D deficiency and rickets. This is due to their dietary intake of the vitamin D being entirely dependent on the nutrient status of their mother. Breastfeeding has been found to be beneficial in terms of infant health and wellbeing. Breast milk is not inherently deficient in vitamin D, but it is directly correlated to maternal vitamin D status, which tends to vary by season, amount of sun exposure, and dietary intake of the various forms of vitamin D [8]. Thus, if the mother has a deficient or low normal vitamin D status, she will not be able to meet the needs of her infant's vitamin D requirements. Additionally, national pediatric and dermatologic societies espouse a general recommendation to keep infants out of the sun as much as possible as a precaution against UV damage. As discussed above, this greatly limits endogenous vitamin production. The incidence of rickets appears greater in infants and children with darker pigmentation because of a 5- to 10-fold greater exposure necessary to produce equal amounts of vitamin D as children with light pigmentation. In general, a daily supplement of 400 IU vitamin D is recommended to address nutritional requirements [7].

## 4. Conclusion

We report two cases of rickets discovered incidentally following Emergency Department visits. Rickets is not a disease of the past, and incidence may be rising secondary due to increased breastfeeding and minimized sun exposure. Laboratory samples and radiographs taken for other reasons may be the first opportunity to diagnose the disease and prevent skeletal deformities and other complications.
